# GRK2 regulates group 2 innate lymphoid cell mobilization in sepsis

**DOI:** 10.1186/s10020-022-00459-8

**Published:** 2022-03-10

**Authors:** Dengming Lai, Weiwei Chen, Kai Zhang, Melanie J. Scott, Yuehua Li, Timothy R. Billiar, Mark A. Wilson, Jie Fan

**Affiliations:** 1 Department of Surgery, University of Pittsburgh School of Medicine, Pittsburgh, 15213 USA; 2 Department of Neonatal Surgery, The Children’s Hospital, Zhejiang University School of Medicine, National Clinical Research Center for Child Health, Hangzhou, 310052 China; 3 McGowan Institute for Regenerative Medicine, University of Pittsburgh, Pittsburgh, PA 15219 USA; 4 Research and Development, Veterans Affairs Pittsburgh Healthcare System, Pittsburgh, PA 15240 USA; 5 Department of Immunology, University of Pittsburgh School of Medicine, Pittsburgh, 15213 USA

**Keywords:** Sepsis, Bone marrow, Mobilization, Group 2 innate lymphoid cells, Aging

## Abstract

**Background:**

Sepsis induces group 2 innate lymphoid cell (ILC2) expansion in the lung. However, the origin of these lung-recruited ILC2 and the mechanism of ILC2 expansion are unclear. This study aims to determine the origin of lung-recruited ILC2 and its underlying mechanism in sepsis.

**Methods:**

Sepsis was induced by cecal ligation and puncture (CLP) model in wild-type, IL-33-deficient and ST2-deficient mice. The frequency, cell number and C-X-C chemokine receptor 4 (CXCR4) expression of ILC2 in bone marrow (BM), blood and lung were measured by flow cytometry. In the in vitro studies, purified ILC2 progenitor (ILC2p) were challenged with IL-33 or G protein-coupled receptor kinase 2 (GRK2) inhibitor, the CXCR4 expression and GRK2 activity were detected by confocal microscopy or flow cytometry.

**Results:**

We show that IL-33 acts through its receptor, ST2, on BM ILC2p to induce GRK2 expression and subsequent downregulation of cell surface expression of CXCR4, which results in decreasing retention of ILC2p in the BM and promoting expansion of ILC2 in the lung. Importantly, we demonstrate that reduced IL-33 level in aging mice contributes to impaired ILC2 mobilization from BM and accumulation in the lung following sepsis.

**Conclusion:**

This study identifies a novel pathway in regulating ILC2p mobilization and expansion during sepsis and indicates BM as the main source of ILC2 in the lung following sepsis.

**Supplementary Information:**

The online version contains supplementary material available at 10.1186/s10020-022-00459-8.

## Introduction

Sepsis, a syndrome of organ dysfunction induced by infection (Singer et al. 2016), is a leading cause of mortality and critical illness worldwide (Fleischmann et al. 2016; Rudd et al. 2020). Despite the advances in intensive care management and therapy, sepsis mortality still remains at a high level, which has been attributed to the alterations in innate and adaptive immune responses (Rubio et al. 2019). Recently, innate lymphoid cells (ILCs) have been identified at barrier surfaces, including lung, skin, and intestine, and found to play an important role in modulating innate and adaptive immunity (Lai et al. 2018). ILCs rapidly respond to inflammation and regulate inflammatory responses by secreting multiple immuno-regulatory cytokines (Golebski 2021; Shannon 2021).

ILC2 is a subgroup of ILCs, suggested to play roles in maintaining airway barrier integrity (Sonnenberg and Artis 2015; Karagiannis 2020), defending against infection (Sugita et al. 2017; Nascimento et al. 2017) and regulating adaptive immunity (Nascimento et al. 2017). In sepsis, ILC2 accumulate at the site of infection, promote polarization of M2 macrophages and enhance T-reg cell expansion (Nascimento et al. 2017). Studies have also suggested that inflammation-induced secretion of cytokine IL-33 regulates ILC2 recruitment and activation. Lung epithelial cells can release IL-33, which in turn mediates ILC2 accumulation leading to epithelial cell responses and airway hyper-reactivity during inflammation (Mohapatra et al. 2016; Moro et al. 2016). In sepsis, ILC2 numbers increase in the lung and regulate immune responses (Nascimento et al. 2017). However, how IL-33 regulates ILC2 recruitment into the lung following sepsis has not been determined. The mechanism underlying ILC2 expansion has been poorly characterized to date, and the origin of sepsis-mobilized ILC2 still remains unknown.

This study aimed to reveal the source of ILC2 and the mechanism of ILC2 accumulation in the lung in sepsis. We used a mouse sepsis model induced by CLP to show that sepsis initiates ILC2p egress from BM in an IL-33/ST2 signaling-dependent manner. We further demonstrate that IL-33, acting through its receptor ST2, induces GRK2 expression and subsequent downregulation of cell surface expression of CXCR4 on BM ILC2p. Decreased CXCR4 expression, in turn, decreases retention of ILC2p in BM, and increases recruitment of ILC2 in the lung during sepsis. Furthermore, reduced IL-33 level in aging mice contributes to impaired ILC2p mobilization from BM, as well as ILC2 recruitment to lung following sepsis. Our findings therefore suggest a novel pathway regulating ILC2p mobilization and expansion during sepsis, and indicate that BM is the main source of recruited ILC2 in the lung.

## Materials and methods

### Mice

Male C57BL/6 J wild-type (WT) mice (6-week-old) and aging C57BL/6 J mice (18-month-old) (The Jackson Laboratories, Bar Harbor, ME), IL-33-deficient (*Il33*^*−/−*^) and ST2-deficient (*St2*^*−/−*^) mice were maintained at the Animal Facility of the University of Pittsburgh School of Medicine. All animal experimental protocols were reviewed and approved by the Institutional Animal Care and Use Committees of University of Pittsburgh and VA Pittsburgh Healthcare System.

CLP was conducted to induced mid-grade sepsis as described previously (Rittirsch et al. 2009). In some experiments, mice were injected intravenously (i.v.) with AMD3100 (3.2 mg/kg B.W. in 100 μl PBS, Tocris Bioscience, Minneapolis, MN) or intraperitoneally (i.p.) with sST2 (0.4 mg/kg B.W. in 200 μl PBS, R&D system, Minneapolis, MN) at 30 min prior to CLP procedure. Some groups of mice were also treated i.v. with recombinant murine IL-33 (40 µg/kg B.W. in 200 μl of PBS, BioLegend, San Diego, CA).

### ILC2 isolation and sorting from bone marrow

BM and lung ILC2 isolation was performed as described before (Moro et al. 2015). In brief, isolated whole lungs were filled with digestion medium [Liberase TM (50 μg/ml, Sigma-Aldrich, St. Louis, MO) and DNase I (1 μg/ml, Sigma-Aldrich)] and digested for 45 min at 37 °C in a water bath. Digested tissue was mashed through 70 μm cell strainers and washed with DMEM media supplemented with 10% FBS, 1% Penicillin/Streptomycin (Thermo Fisher Scientific, Pittsburgh, PA). For BM ILC2p isolation, BM cells were flushed from femurs of mice with DMEM, and passed through 70 μm cell strainers. Red blood cells were lysed by RBC lysis buffer (Thermo fisher Scientific). For BM ILC2p sorting, total BM cells were stained with lineage cocktail Abs, anti-ST2 Ab (DJ8, MD Biosciences, Oakdale, MN), anti-CD90.2 Ab (53–2.1, eBioscience, San Diego, CA), anti-KLRG1 Ab (2F1, BD Biosciences, San Jose, CA) and anti-Sca-1 Ab (D7, BD Biosciences), at 4 °C for 30 min. Lin^−^CD90.2^+^ ST2^+^KLRG1^−^ cells or Lin^−^CD90.2^+^ST2^+^CD45^+^ cells were sorted by FACSAria (BD Biosciences). The purity of ILC2p was determined as > 98% and the cell viability was > 95%.

### Flow cytometry

Cell suspensions were stained with antibodies for mouse studies as follows: FITC-conjugated B220 (RA3-6B2, eBioscience), CD3 (17A2, eBioscience), CD4 (RM4-5, eBioscience), CD5 (53–7.3, eBioscience), CD8α (53–6.7, eBioscience), CD11b (M1/70, eBioscience), CD11c (N418, eBioscience), CD19 (eBio1D3, eBioscience), Gr-1 (RB6-8C5, eBioscience), TCRβ (N57-597, eBioscience), Ter-119 (Ter119, eBioscience), γδTCR (eBioGL3, eBioscience), NK1.1 (PK136, eBioscience), FcεR1 (MAR-1, eBioscience). PE-conjugated ST2 (DJ8, MD Biosciences), PerCP/Cy5.5-cinjugated CXCR4 (Biolegend). APC-conjugated CD90.2 (53–2.1, eBioscience). Alexa Fluor 700-conjugated CD45 (30-F11, eBioscience). BV421-conjugated Ly-6A/E (D7, BD Biosciences). PE-Cyanine 7-conjugated KLRG1 (2F1, BD Biosciences).

To stain GRK2 antigen (R&D systems), cells were fixed and permeabilized after cell surface marker staining. Cells were analyzed by BD LSRII flow cytometer (BD Bioscences) and FlowJo software.

### ELISA

IL-33 in plasma was detected by ELISA (eBioscience) according to manufacturer’s instructions.

### Immunofluorescence staining

Sorted BM ILC2 were fixed with 4% paraformaldehyde for 20 min and permeabilized with 0.01% Triton X-100 for 20 min. After washing, cells were incubated with primary rabbit antibody to GRK2 (R&D systems) at 4 °C overnight and secondary Alexa Fluor 488 conjugated antibody to rabbit for 1 h at room temperature. After that, cells were stained with Hoechst 33258 (Sigma-Aldrich) and GRK2 antibody (R&D system) and measured by confocal microscopy (Olympus, Fluoview-FV1000, Olympus America Co., Center Valley, PA).

### Statistical analysis

GraphPad Software was applied to analyze data by two-tailed unpaired Student’s t-tests or one-way ANOVA tests by Brown-Forsythe test. Data are presented as mean ± SEM. *P* < 0.05 was considered statistically significant. (*, *P* < 0.05; **, *P* < 0.01; NS = not significant).

## Results

### ILC2 egress from bone marrow following sepsis

**Fig. 1:**
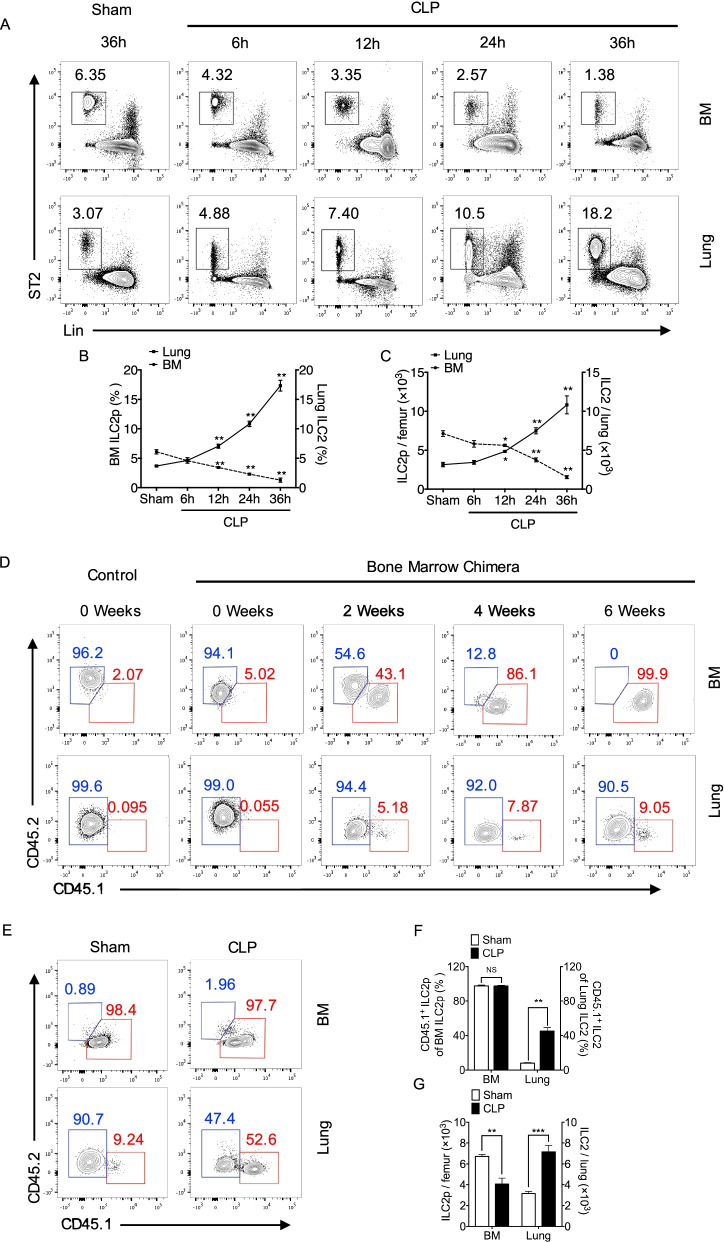
ILC2 egress from BM following sepsis. **A** Representative flow cytometry plots showing percentages of ILC2p in BM and ILC2 in lung of WT mice after sham surgery (36 h) and at 6, 12, 24, and 36 h after CLP (n = 5 mice/group). **B** Line graph showing BM ILC2p (left Y-axis) and lung ILC2 (right Y-axis) frequency at time points up to 36 h after CLP (n = 5 mice/group). **C** The absolute number of BM ILC2p (left Y-axis) and lung ILC2 (right Y-axis) at time points up to 36 h after CLP (n = 5 mice/group). **D** Representative flow cytometry plots showing frequencies of CD45.1 and CD45.2 of ILC2 in bone marrow and lung from bone marrow chimera mice (n = 3 mice/group). **E** Representative flow cytometry plots showing frequencies of CD45.1 and CD45.2 of ILC2 in bone marrow and lung from bone marrow chimera mice at 24 h after sham surgery or CLP (n = 4–5 mice/group). **F** The frequencies of CD45.1^+^ ILC2 in bone marrow (left Y-axis) and lung (right Y-axis) at 24 h after CLP (n = 4–5 mice/group). **G** The absolute number of BM ILC2p (left Y-axis) and lung ILC2 (right Y-axis) at 24 h after CLP in chimera septic mice (n = 4–5 mice/group). Data are shown as mean ± SEM. * *P* < 0.05, ** *P* < 0.01, NS = not significant

Sepsis was induced in WT mice using CLP. ILC2p in the BM and ILC2 in the lung at the time points up to 36 h after CLP were detected by flow cytometry. BM ILC2p were defined as Lin^−^CD90.2^+^ST2^+^ KLRG1^−^ cells, and lung ILC2 was defined as Lin^−^CD90.2^+^ST2^+^CD45^+^ cells (Additional file 1: Figure S1A & B) (Seehus et al. 2015). We found that with the increase in the lung ILC2 following CLP, BM ILC2p significantly decreased (Fig. 1A–C). These results suggest a negative correlation between increased ILC2 in the lung and decreased ILC2p in BM. Moreover, we detected that ILC2 accumulated in circulating blood at 24 h and 36 h (Additional file 1: Figure S2B–D). This response prompted that BM ILC2p migrated to lung tissue through peripheral blood.

To further confirm that ILC2p from BM is the major source of recruited ILC2 in the lung, we created CD45.2 and CD45.1 chimera mice (Additional file 1: Fig. S1C), in which CD45.1 ILC2 nearly 100% replaced CD45.2 ILC2 in the BM at 6 weeks after chimera procedure (Fig. 1D). In chimera septic mice, the number of ILC2 in the lung is about twofold greater than that in sham animals, however, ILC2p in BM are decreased at 24 h after CLP, which is similar to the changes seen in non-chimera mice (Fig. 1G); and, notably, CD45.1 ILC2 in chimera septic mice occupied ~ 52% of total ILC2 in the lung, whereas, CD45.1 ILC2 in chimera sham animals are about 10% of total ILC2 in the lung. These results reveal that nearly 100% lung-recruited ILC2 are from bone marrow (Fig. 1E & F).

### IL-33 is required for ILC2 egress from bone marrow

**Fig. 2:**
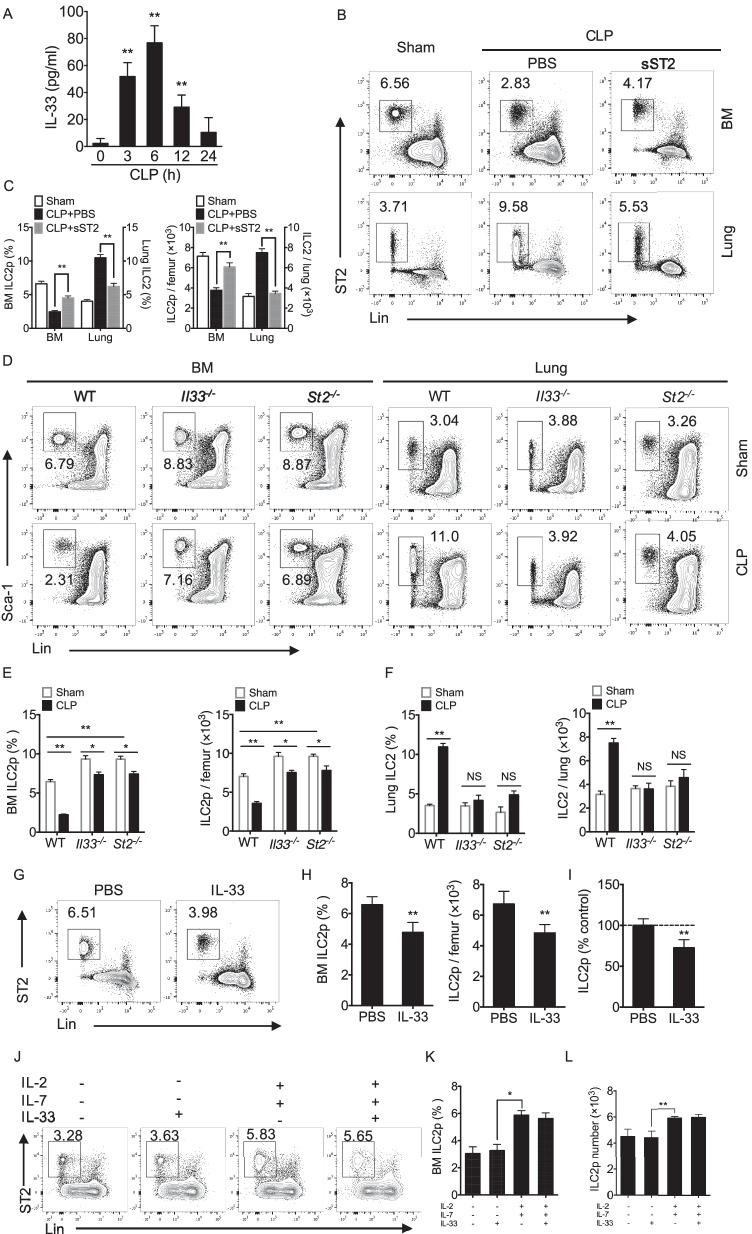
IL-33 is required for ILC2 egress from BM. **A** Plasma IL-33 in WT mice at time points up to 24 h after CLP (n = 3–5 mice/group). **B** Representative flow cytometry plots and **C** bar graphs showing percentages of BM ILC2p and lung ILC2 in WT mice after PBS or recombinant murine sST2 injection (10 μg sST2 in 200 μl PBS) 30 min prior to CLP. After 24 h, lung and BM were collected for flow analysis (n = 5 mice/group). **D** Representative flow cytometry plots and bar graph of BM ILC2p (**E**) and lung ILC2 (**F**) percentages and absolute number of WT, *Il33*^*−/−*^, *St2*^*−/−*^ mice at 24 h after CLP (n = 3–5 mice/group). **G** Flow cytometry of BM ILC2p from mice treated with PBS or rmIL-33 (40 µg/kg B.W, i.v) for 24 h (n = 5 mice/group). **H** BM ILC2p percentage and absolute number in PBS or rmIL-33-treated mice (n = 5 mice/group). **I** BM ILC2p number in control mice were normalized as 100%. The percentage of remaining BM ILC2p cells after IL-33 treatment in control mice was analyzed. **J** Bone marrow cells were treated with/without IL-2 (10 ng/ml), IL-7 (10 ng/ml) and IL-33 (50 ng/ml), and frequencies of ILC2p were analyzed by flow cytometry. **K** The frequencies of ILC2p in bone marrow at 24 h after stimulation of IL-2, IL-7 or IL-33 (n = 3 per group). **L** The absolute number of ILC2p in bone marrow at 24 h after stimulation of IL-2, IL-7 or IL-33 (n = 3 per group). Data are shown as mean ± SEM. *P < 0.05, **P < 0.01

Recent study showed that IL-33 mediates ILC2 recruitment and activation (Matsuyama 2021). However, the role of IL-33 in ILC2 egression from BM in sepsis remains unclear. To elucidate the role of IL-33 in mediating ILC2 mobilization following sepsis, we measured IL-33 protein concentrations in the blood up to 24 h after CLP. We found that CLP significantly increased plasma IL-33 level, which peaked at 6 h, and decreased by 24 h (Fig. 2A). To determine the role for IL-33 in mediating sepsis-induced ILC2 egression, we intraperitoneally injected WT mice with recombinant mouse soluble ST2 (sST2), the soluble form of IL-33 receptor, at 30 min prior to CLP. sST2 binds to IL-33 and reduces IL-33 availability to bind and signal through cell surface ST2. Administration of sST2 significantly decreased ILC2p egress from BM into the lung at 24 h after CLP (Fig. 2B & C).

Furthermore, we subjected *Il33*^*−/−*^ and *St2*^*−/−*^ mice to CLP for 24 h, and then assessed the changes in ILC2 in the BM and lung. In *St2*^*−/−*^ mice, we used Sca-1, instead of ST2, as a marker for defining ILC2 based on the facts, as shown in Additional file 1: Fig. S1A, that (1) more than 95% of CD90.2^+^Lin^−^ST2^+^ cells or CD90.2^+^Lin^−^ST2^+^KLRG1^−^ cells express Sca-1; and (2) more than 95% of CD90.2^+^Lin^−^Sca-1^+^ cells express ST2. Therefore, we defined BM ILC2p as CD90.2^+^Lin^−^Sca-1^+^ cells. Similarly, in the lung, more than 90% of Lin^−^CD90.2^+^ ST2^+^CD45^+^ cells express Sca-1 and over 90% of CD45^+^CD90.2^+^Lin^−^Sca-1^+^ cells express ST2 (Additional file 1: Figure S1B). Thus, we defined lung ILC2 as CD45^+^CD90.2^+^Lin^−^Sca-1^+^ cells (Nascimento et al. 2017; Moro et al. 2015). As shown in Figs. 2D & E, genetic deficiency of either IL-33 or ST2 prevented ILC2p egression from BM following sepsis. In addition, administration of IL-33 to WT sham mice significantly promoted egress of BM ILC2p (Fig. 2G–I). In order to address whether IL-33 promotes ILC2 migration and/or proliferation, we determine the effect of IL-33 on ILC2p proliferation. We found that IL-33 failed to induce ILC2p proliferation, while IL-2 and IL-7 are able to increase the number of ILC2p (Fig. 2J–L). Collectively, these results indicate that IL-33/ST2 signaling promotes ILC2 egress from BM following sepsis.

### IL-33 induces ILC2 egress through down-regulation of CXCR4 expression

**Fig. 3:**
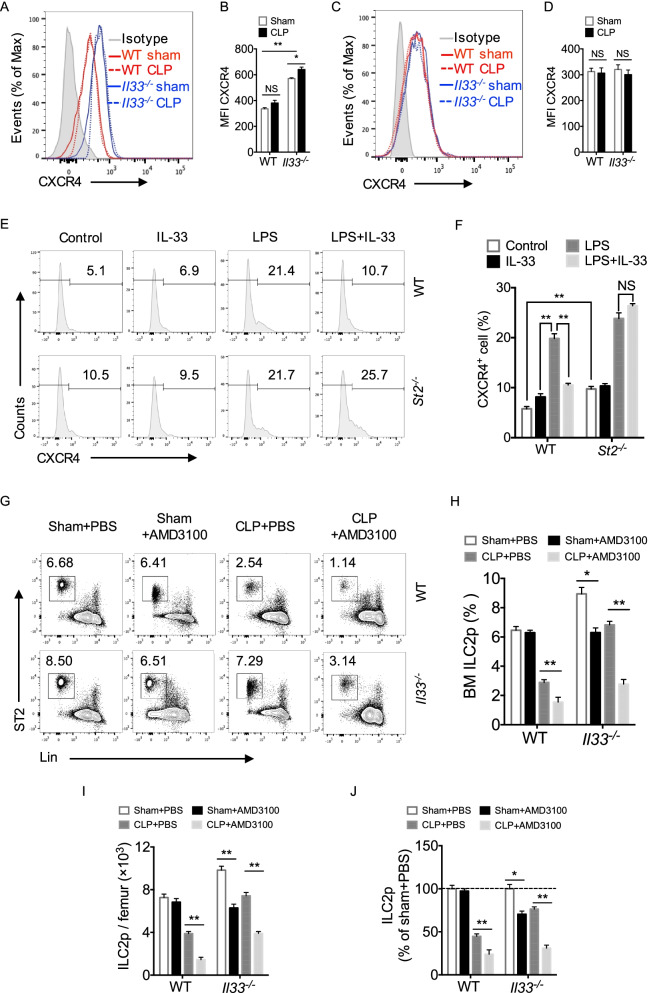
IL-33 induces ILC2 egress through down-regulating CXCR4. **A** Expression and **B** mean fluorescence intensity (MFI) of CXCR4 on BM ILC2p from WT and *Il33*^*−/−*^ mice in response to sham surgery or CLP for 24 h analyzed by flow cytometry (n = 4–5 mice/group). **C** Expression and **D** MFI of CXCR4 on lung ILC2 from WT and *Il33*^*−/−*^ mice after sham surgery or CLP for 24 h (n = 4–5 mice/group). **E**, **F** Expression of CXCR4, shown by flow cytometry and bar graph, on BM ILC2p at 24 h after LPS (1 μg/ml) with or without rmIL-33 (50 ng/ml) treatment and IL-2 (10 ng/ml), IL-7 (10 ng/ml) (n = 3 per group). **G** Representative flow cytometry plots and **H** & **I** bar graphs showing BM ILC2p percentages and absolute number of Sham + PBS, sham + AMD3100, CLP + PBS, and CLP + AMD3100 in WT, *Il33*^*−/−*^ mice. PBS (100 μl) or AMD3100 [3.2 mg/kg B.W. in 100 μl PBS] were injected intravenously (i.v.) 30 min prior to CLP, and CLP for 24 h (n = 4–5 mice/group). **J** The percentage of number in each group to sham surgery mice treated with PBS. Number in BM of sham + PBS mice normalized to 100%. Data shown are the mean ± SEM. *P < 0.05, **P < 0.01, NS = not significant

CXCR4 signaling plays important roles in the retention of lymphoid cells, stem cells, and progenitor cells in the BM (Beck et al. 2014; Pitchford et al. 2009). A recent study showed that CXCR4 is also involved in the regulation of ILC2p mobilization from the BM (Stier 2017). To determine the role of CXCR4 signaling in ILC2p egress from the BM following sepsis, we first measured cell surface expression of CXCR4 on ILC2p in BM from WT and *Il33*^*−/−*^ mice. We found that the cell surface expression of CXCR4 was increased in *Il33*^*−/−*^ ILC2p as compared to that in WT ILC2p. At 24 h after CLP, cell surface expression of CXCR4 was not changed in WT ILC2p but was increased in *Il33*^*−/−*^ ILC2p after CLP (Fig. 3A & B). In contrast, there was no significant difference in cell surface expression of CXCR4 on lung ILC2 between WT and *Il33*^*−/−*^ mice at 24 h after either sham or CLP surgery (Fig. 3C & D). These observations were recapitulated using an i*n vitro* assay. As shown in Fig. 3E and F, LPS stimulation of WT ILC2p (1 μg/ml, for 24 h) significantly increased CXCR4 surface expression on ILC2p. Pretreatment with rmIL-33 (50 ng/ml) significantly reduced the CXCR4 cell surface expression on WT ILC2p in response to LPS, but failed to decrease the CXCR4 cell surface expression on *St2*^*−/−*^ ILC2p. These data suggest that IL-33 acting through ST2 down-regulates ILC2 surface expression of CXCR4 following LPS stimulation.

To further confirm the role of CXCR4 in regulating ILC2 mobilization, we pretreated WT and *Il33*^*−/−*^ mice with CXCR4 antagonists (AMD3100, 3.2 mg/kg B.W. i.v.) at 30 min before CLP, and measured BM ILC2p at 24 h after CLP. The treatment of CXCR4 antagonist further decreased ILC2p in the BM in WT mice following CLP, and this response associated with increased ILC2p egress from BM (Fig. 3G & H). In *Il33*^*−/−*^ mice, CLP induced less egress of BM ILC2p, but CXCR4 antagonism reversed effects of IL-33 deficiency and promoted ILC2p egress from the BM (Fig. 3G–J). The data demonstrate that IL-33 deficiency results in largely retained ILC2p in BM, while inhibition of CXCR4 promotes ~ 70% of ILC2p to egress from the BM (Fig. 3J). Taken together these data support that IL-33/ST2-downregulated CXCR4 expression promotes ILC2p egress from BM.

### GRK2 mediates LPS- and IL-33-modulated CXCR4 expression

**Fig. 4:**
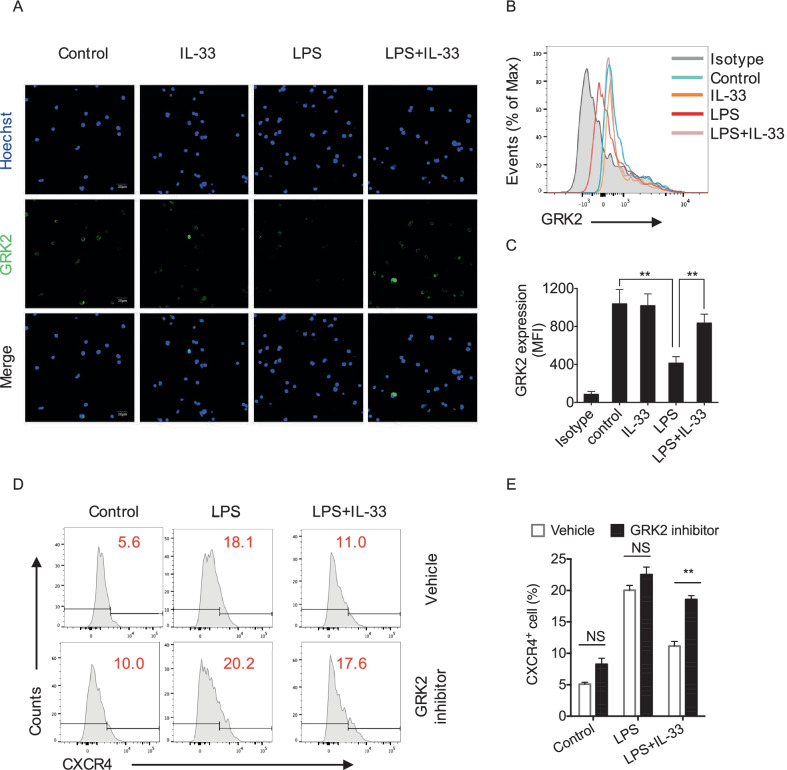
GRK2 mediates IL-33/ST2 signaling-induced decrease in CXCR4. **A)** Confocal microscopy immunofluorescence of GRK2 (green) and Hoechst (blue) in BM ILC2p at 1 h after stimulation with LPS (1 μg/ml) and/or rmIL-33 (50 ng/ml) and IL-2 (10 ng/ml), IL-7 (10 ng/ml) (n = 3 per group). **B** Flow cytometric analysis and **C** MFI of GRK2 expression in BM ILC2p after treatment with LPS and/or rmIL-33 for 1 h (n = 3 per group). **D** & **E** The percentages of CXCR4 expressing cells in total BM ILC2p after LPS (1 μg/ml) with or without rmIL-33 (50 ng/ml) or GRK2 inhibitor (150 μM) stimulation for 24 h (n = 3 per group). Data is representative of three independent in vitro experiments. Data are shown as the mean ± SEM, *P < 0.05, **P < 0.01

GRKs are serine-threonine protein kinases that induce internalization of G-protein coupled receptors, including chemokine receptors, e.g. CXCR2 and CXCR4 (Liu et al. 2013; Fan and Malik 2003). We therefore investigated whether GRKs play a role in regulating CXCR4 cell surface expression on BM ILC2p in sepsis. Firstly, we detected expression levels of four widely distributed GRKs including GRK2, GRK3, GRK5, and GRK6 in ILC2p at 1 h after LPS or IL-33 treatment using confocal microscopy. We found that only GRK2 expression changed in this experimental setting. At 1 h, GRK2 expression in LPS-treated ILC2p was attenuated, and GRK2 expression was restored by IL-33 in the LPS-treated ILC2p (Fig. 4A). We further measured GRK2 expression by flow cytometry in ILC2p isolated from BM. As shown in Fig. 4B and C, ILC2p treated with LPS demonstrated low expression of GRK2, and IL-33 treatment restored GRK2 expression in ILC2p in LPS-treated ILC2p, although IL-33 alone did not affect the GRK2 expression. Importantly, inhibition of GRK2 by GRK2 inhibitor (βARK1, 150 µM) reversed IL-33-induced downregulation of CXCR4 (Fig. 4D & E). These data suggest that GRK2 mediates LPS- and IL-33-modulated CXCR4 expression.

### Reduced IL-33 in aging mice attenuates ILC2p mobilization in sepsis

**Fig. 5:**
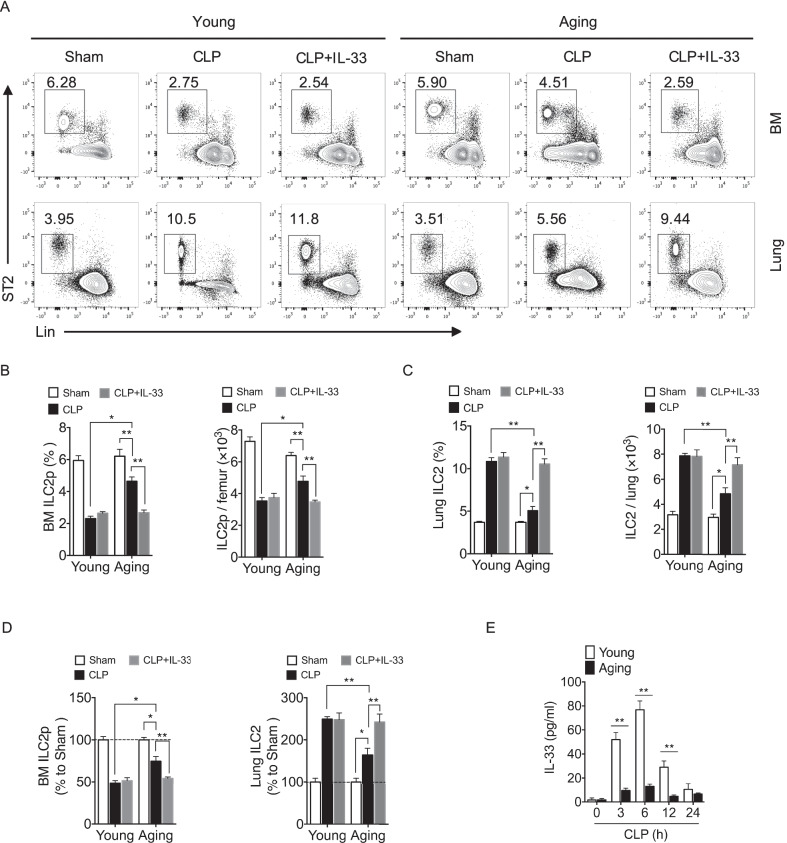
Reduced IL-33 in aging mice attenuates ILC2p mobilization in sepsis. **A** Representative flow cytometry plots showing percentages of ILC2p in BM and ILC2 in lung of young and aging mice. Frequency and number of (**B**) BM ILC2p and **C** lung ILC2 at 24 h after sham, CLP and CLP + IL-33 (n = 4–5 mice/group). **D** Percentages of retained ILC2p in BM or ILC2 recruited in lung of mice treated with IL-33 (40 µg/kg B.W.) intravenously 30 min before CLP compared to sham surgery mice. BM ILC2p or lung ILC2 in sham surgery were normalized to 100% (n = 4–5 mice/group). **E** IL-33 in plasma of aging mice compared to young mice after CLP for up to 24 h (n = 4–5 mice/group). Data shown as mean ± SEM. * *P* < 0.05, ** *P* < 0.01

Sepsis-induced innate and adaptive immune system dysfunction leads to mortality and poor outcomes (Delano and Ward 2016; Hotchkiss et al. 2013). The incidence of sepsis is much higher in elderly adults, and older septic patients have worse outcomes (Rudd et al. 2020; Weng et al. 2018). We found that in aging mice (18-month-old) sepsis-induced ILC2 mobilization from BM and expansion in lung were significantly decreased compared to young mice (6-week-old) (Fig. 5A & B). Our data show that there are more ILC2p retained in BM of aging mice at 24 h after CLP. The percentage and number of ILC2p in BM of aging mice were higher compared to young mice (Fig. 5B & C). Consistent with reduced ILC2p mobilization in aging mice in response to CLP, recruitment of ILC2 in the lung was also decreased (Fig. 5B & C). In addition, the mobilized ILC2p in aging mice were about ~ 30% of total ILC2p cells as compared to ~ 60% in young mice (Fig. 5D). We further found that IL-33 levels in peripheral plasma of aging animals is significantly lower than in young animals after CLP (Fig. 5E). Interestingly, treatment of aging mice with exogenous IL-33 (40 µg/kg B.W.) at the time of CLP procedure significantly decreased BM ILC2p and increased lung ILC2 at 24 h after CLP (Fig. 5A–D). Taken together, reduced IL-33 production in aging mice impaired ILC2p mobilization from BM in sepsis.

## Discussion

A number of studies have demonstrated that ILC2 regulate lung inflammation and promote lung tissue homeostasis, and that functional ILC2 accumulation in the lung of sepsis (Krishack, et al. 2019). The current study shows that sepsis leads to a significant mobilization of ILC2p from the BM and accumulation in the lung. The egress requires IL-33/ST2 signaling, leading to downregulation of CXCR4 expression on the surface of ILC2p, which serves as a major retention force keeping ILC2p in the BM (Stier 2017). GRK2 plays an important role in mediating IL-33/ST2 signaling and downregulation of CXCR4, which promotes ILC2 egress from BM. Moreover, reduced lung ILC2 expansion in aging mice was attributed to the reduced IL-33 levels and impaired ILC2p mobilization from the BM. This study, therefore, explores BM as a main source of peripheral ILC2 in sepsis and a novel pathway that promotes ILC2 mobilization from BM.

Hematopoietic cell mobilization from the BM, triggered by infectious signals via Toll-like receptors (TLR) or non-TLR pathways, is required for sepsis survival and recovery (Liu et al. 2013; Delano et al. 2011). Studies suggest that ILC2 are differentiated from common lymphoid progenitors in BM and enriched in barrier tissue (Lai et al. 2016). Some studies revealed that the accumulated ILC2 in lung was derived from local development and other tissue site (Huang et al. 2018; Bai, et al. 2021). However, the source of peripheral ILC2 in a pathological condition and the mechanisms involved in ILC2 mobilization and accumulation remain to be fully addressed (Ricardo-Gonzalez et al. 2018). In this study, we reveal the decreased ILC2p in the BM with increased ILC2 in the lung. These coordinate alterations in ILC2 in BM and lung tissue occur in a dynamic manner. In order to determine the source of migrated ILC2 in the lung, we established the CD45.1 and CD45.2 chimera mice to track the ILC2 from BM. We found that ILC2 released from BM is the major source of the lung ILC2 in response to sepsis.

IL-33 is known to promote lung ILC2 recruitment and activation during inflammation (Gadani et al. 2017). Recently, IL-33 has also been reported to promote ILC2p mobilization (Stier 2017). However, the role of IL-33 in ILC2 egression in sepsis has yet been studied. In this study, we show that systemic IL-33 levels increase in response to sepsis, and increased IL-33 is required for ILC2p egress from BM and expansion in the lung. Neutralization of IL-33 with sST2 significantly reduced BM ILC2p egress and lung ILC2 expansion, and administration of IL-33 induces BM ILC2p mobilization. Furthermore, genetic deficiency of IL-33 and ST2 prevented sepsis-induced decreases in ILC2p in the BM and increases in the lung. These data strongly suggest that BM ILC2 mobilization and emigration require IL-33/ST2 signaling.

CXCR4 signaling is one of the important factors governing hematopoietic cell retention in the BM. CXCR4 signaling has been reported as a cell-extrinsic mechanism controlling egress of immature B lymphocyte and maintenance of stem cells, ILC2p and progenitor cells in the BM (Beck et al. 2014; Stier 2017). In the current study, we investigated the interaction between IL-33/ST2 signaling and CXCR4 expression in ILC2p and observed an IL-33/ST2-induced downregulation of CXCR4 cell surface expression on ILC2p, and therefore, a reduction of ILC2p retaining in BM. By comparing the CXCR4 expression in BM ILC2p and lung ILC2, as shown in Fig. 3B and D, we can see that the expression of CXCR4 in BM ILC2p is higher than that in lung ILC2. Since the ILC2 mobilization from BM is a dynamic course, the group of ILC2p with decreased CXCR4 would quickly egress from BM, which resulted in the disappear of the ILC2p with high expression of CXCR4 in the BM following CLP. Moreover, we found that exogenous IL-33 prevents the increase in CXCR4 cell surface expression on ILC2 following LPS stimulation, and downregulation of CXCR4 depends on ST2 signaling. Administration of AMD3100, a CXCR4 antagonist, largely induces ILC2 egress, which is blocked in IL-33 deficient mice during sepsis. However, LPS-stimulation induces increased CXCR4 in BM ILC2p. Co-stimulation with LPS and IL-33 also increased expression of CXCR4 compared to control. As reported previously, IL-2 significantly increases CXCR4 expression on BM ILC2p (Stier 2017). This may explain the differences in CXCR4 expression on BM ILC2p in vivo and in vitro, and demonstrates that LPS stimulation alone may not induce BM ILC2p mobilization. Thus, these data explore a mechanism of sepsis-induced ILC2 egress from BM through IL-33-mediated down-regulation of CXCR4 expression on ILC2, thereby enhancing ILC2 release.

Regulation of CXCR4 includes the transcriptional control, protein expression, receptor internalization, and degradation (Busillo and Benovic 2007). GRK2 promotes CXCR4 internalization (Jimenez-Sainz et al. 2006). As we demonstrated, IL-33 negatively regulates CXCR4 after LPS stimulation. However, the downregulation of CXCR4 by IL-33 was prevented by GRK2 inhibitor, and in LPS-treated ILC2p, GRK2 expression was restored by IL-33. Thus, these results reveal a novel pathway of CXCR4 regulation whereby IL-33 negatively regulates CXCR4 by promoting GRK2 expression. It has been reported in some studies that IL-33 facilitates bacterial clearance by regulating neutrophil-mediated bactericidal, which may alter the course of sepsis (Alves-Filho et al. 2010; Robinson et al. 2018). In our in vitro bacteria-free study, we observed that IL-33 acting through ST2 receptor prevents GRK2 induction and therefore, maintains CXCR4 expression in ILC2p in a condition lacking bacteria challenge. Thus, we infer that IL-33 restores GRK2 through ST2 signaling rather than IL-33-promoted bacterial clearance. GRK2 plays a central role in the regulation of GPCR desensitization. GRK2 can be downregulated by LPS, and then the internalization of CXCR4 in ILC2 is attenuated, which results in restraint of ILC2p in the bone marrow. Once IL-33 was added to the culture medium, GRK2 expression in ILC2 was restored. The questions remain to be fully addressed include how GRK2 expression was restored by IL-33 and how TLR4 signaling is intervening in the IL-33/ST2 signaling in the egress of ILC2p from BM?

Age is an independent predictor of sepsis mortality (Rudd et al. 2020; Weng et al. 2018). Age alters immune responses following sepsis compared to young mice. However, the ILC2 response to sepsis in aging is unclear. In the current study, we showed reduced ILC2 recruitment in the lungs of aging mice. This reduction is attributed to impaired BM ILC2p mobilization. As we demonstrated in the results, IL-33 plays a critical role in BM ILC2p mobilization. Plasma IL-33 levels significantly increased in young mice after CLP. However, the levels of IL-33 were much lower in aging mice. Thus, our findings suggest that reduced IL-33 response in sepsis in aging mice attenuates BM ILC2p mobilization and lung ILC2 recruitment. Our previous study showed that ILC2 plays an important role in protecting lung endothelial cell from pyroptosis in sepsis-induced acute lung inflammation (Lai et al. 2018). Thus, reduced ILC2 mobilization and egression in aged septic mice might serve as a detrimental fact contributes to higher mortality and morbidity.

## Conclusions

In summary, sepsis induces ILC2p egress from BM and this egress requires downregulation of CXCR4 expression by IL-33 during sepsis. IL-33 maintains GRK2 levels, which mediate CXCR4 downregulation after LPS stimulation. Reduction of ILC2 mobilization in septic aging mice results in impaired lung ILC2 recruitment. This study defines a novel mechanism underlying ILC2 mobilization in sepsis and a new therapeutic target of ILC2 regulation in sepsis.

## Supplementary Information

**Additional file 1: Figure S1.** Flow cytometry strategy of ILC2 and strategy of chimera mice. **(A)** BM ILC2p were defined as CD90.2^+^Lin^−^ST2^+^KLRG1^−^ cells. A lymphocyte gate was drawn and CD90.2^+^ cells were gated into Lin^−^ST2^+^ and Lin^−^Sca-1^+^ cells. Flow cytometry plots show expression of Sca-1 in ILC2p and ST2 in CD90.2^+^Lin^−^Sca-1^+^ cells. **(B)** Lung ILC2 were defined as CD90.2^+^CD45^+^ Lin^−^ST2^+^ cells. A lymphocyte gate was drawn, and then CD90.2^+^CD45^+^ cells were gated into Lin^−^ST2^+^. The expression of Sca-1 on CD90.2^+^CD45^+^Lin^−^ST2^+^ cells and ST2 expression on CD90.2^+^CD45^+^Lin^−^Sca-1^+^ cells were detected. **(C)** 6-week old CD45.2 mice were irradiated by RS2000 pro (8 Gy), and reconstituted with 10^7^ cells of CD45.1 WT bone marrow cells. The bone marrow and lung tissue were harvested from the chimera mice at 0, 2, 4, and 6 weeks after bone marrow reconstitution. **Figure S2.** ILC2 in blood following sepsis of mice. (A) Representative flow cytometry plots and gating strategy of peripheral blood ILC2. ILC2 was defined as CD90.2 + CD45 + Lin-ST2 + cells. A lymphocyte gate was drawn, and then CD90.2 + CD45 + cells were gated into Lin-ST2 + . (B) Representative flow cytometry plots showing percentages of ILC2 in peripheral blood of WT mice after sham surgery (36 h) and 6, 12, 24, and 36 h after CLP. (C) Line graph showing blood ILC2 frequency at time points up to 36 h after CLP. N > 5 mice/group. (D) The absolute numbers of blood ILC2 at time points up to 36 h after CLP were calculated in every 1 × 106 CD45 + cells. Data shown as mean ± SEM. NS = not significant, *P < 0.05, **P < 0.01.

## Data Availability

All data generated or analyzed during this study are included in this published article and its Additional file 1. The original data be made available for assessment upon request by the editors.

## Abbreviations

BM: Bone marrow
B.W.: Body weight
CLP: Cecal ligation and puncture
CXCR4: C-X-C Motif Chemokine Receptor 4
GRK2: G protein-coupled receptor kinase-2
IL-33: Interleukin 33
ILC2: Group 2 innate lymphoid cells
ILC2p: ILC2 progenitor
ILCs: Innate lymphoid cells
i.p.: Intraperitoneal
i.v.: Intravenous
KLRG1: Killer cell lectin-like receptor subfamily G member 1
LPS: Lipopolysaccharide
Sca-1: Stem cells antigen-1
sST2: Soluble ST2
TLR: Toll-like receptors
WT: Wild type
