# Supplementary material for: GRK2 regulates group 2 innate lymphoid cell mobilization in sepsis
Source: Mol Med. 2022 Mar 10;28:32. doi: 10.1186/s10020-022-00459-8 (PMC8908620; doi:10.1186/s10020-022-00459-8)
Supplement: Supplementary file 1 — Additional file 1: Figure S1. Flow cytometry strategy of ILC2 and strategy of chimera mice. (A) BM ILC2p were defined as CD90.2+Lin−ST2+KLRG1− cells. A lymphocyte gate was drawn and CD90.2+ cells were gated into Lin−ST2+ and Lin−Sca-1+ cells. Flow cytometry plots show expression of Sca-1 in ILC2p and ST2 in CD90.2+Lin−Sca-1+ cells. (B) Lung ILC2 were defined as CD90.2+CD45+ Lin−ST2+ cells. A lymphocyte gate was drawn, and then CD90.2+CD45+ cells were gated into Lin−ST2+. The expression of Sca-1 on CD90.2+CD45+Lin−ST2+ cells and ST2 expression on CD90.2+CD45+Lin−Sca-1+ cells were detected. (C) 6-week old CD45.2 mice were irradiated by RS2000 pro (8 Gy), and reconstituted with 107 cells of CD45.1 WT bone marrow cells. The bone marrow and lung tissue were harvested from the chimera mice at 0, 2, 4, and 6 weeks after bone marrow reconstitution. Figure S2. ILC2 in blood following sepsis of mice. (A) Representative flow cytometry plots and gating strategy of peripheral blood ILC2. ILC2 was defined as CD90.2 + CD45 + Lin-ST2 + cells. A lymphocyte gate was drawn, and then CD90.2 + CD45 + cells were gated into Lin-ST2 + . (B) Representative flow cytometry plots showing percentages of ILC2 in peripheral blood of WT mice after sham surgery (36 h) and 6, 12, 24, and 36 h after CLP. (C) Line graph showing blood ILC2 frequency at time points up to 36 h after CLP. N > 5 mice/group. (D) The absolute numbers of blood ILC2 at time points up to 36 h after CLP were calculated in every 1 × 10^6^ CD45 + cells. Data shown as mean ± SEM. NS = not significant, *P < 0.05, **P < 0.01. [file 10020_2022_459_MOESM1_ESM.docx]

**Supplementary Information**


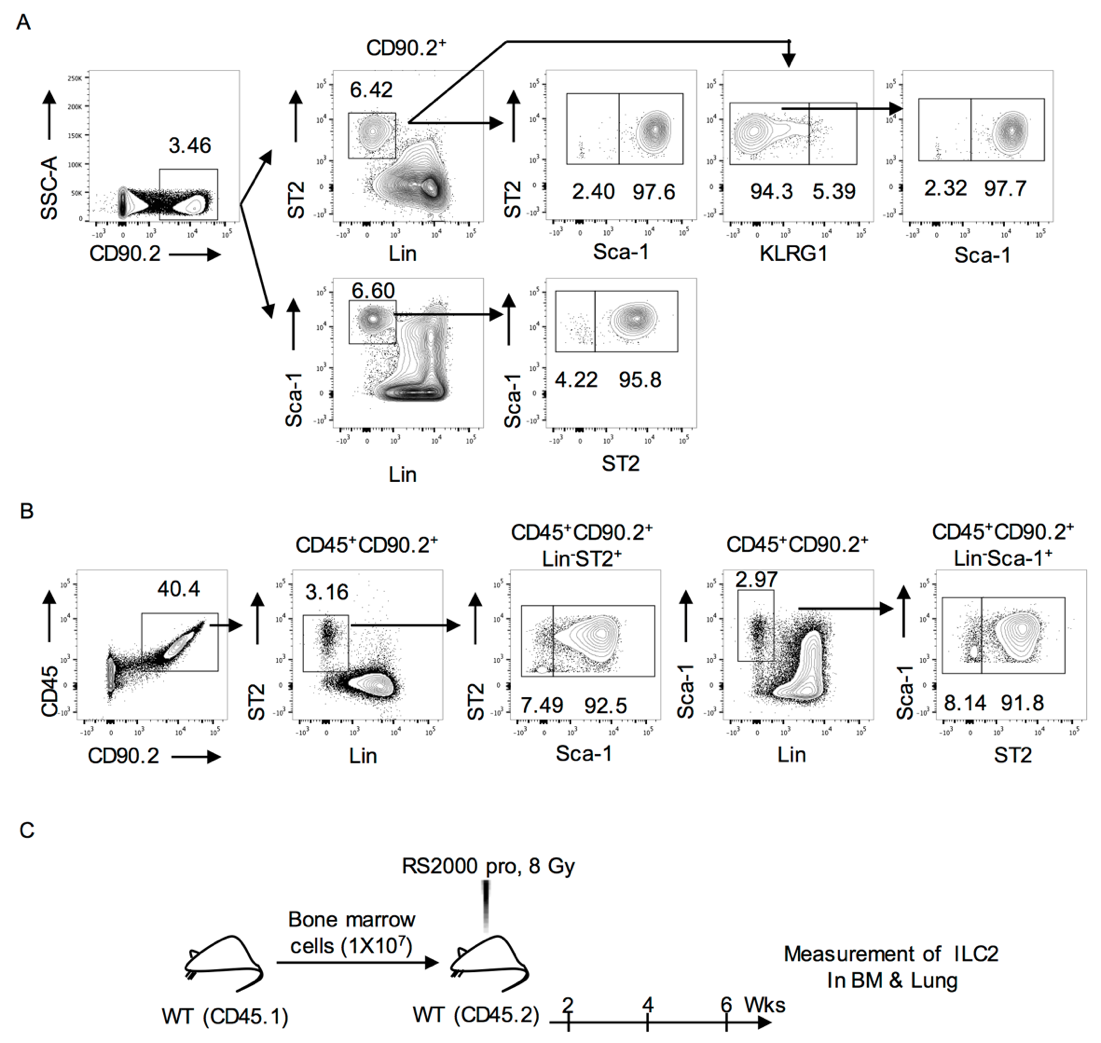


**Supplementary Figure E1. Flow cytometry strategy of ILC2 and strategy of chimera mice.**

**(A)** BM ILC2p were defined as CD90.2^+^Lin^-^ST2^+^KLRG1^-^ cells. A lymphocyte gate was drawn and CD90.2^+^ cells were gated into Lin^-^ST2^+^ and Lin^-^Sca-1^+^ cells. Flow cytometry plots show expression of Sca-1 in ILC2p and ST2 in CD90.2^+^Lin^-^Sca-1^+^ cells. **(B)** Lung ILC2 were defined as CD90.2^+^CD45^+^ Lin^-^ST2^+^ cells. A lymphocyte gate was drawn, and then CD90.2^+^CD45^+^ cells were gated into Lin^-^ST2^+^. The expression of Sca-1 on CD90.2^+^CD45^+^Lin^-^ST2^+^ cells and ST2 expression on CD90.2^+^CD45^+^Lin^-^Sca-1^+^ cells were detected. **(C)** 6-week old CD45.2 mice were irradiated by RS2000 pro (8 Gy), and reconstituted with 10^7^ cells of CD45.1 WT bone marrow cells. The bone marrow and lung tissue were harvested from the chimera mice at 0, 2, 4, and 6 weeks after bone marrow reconstitution.


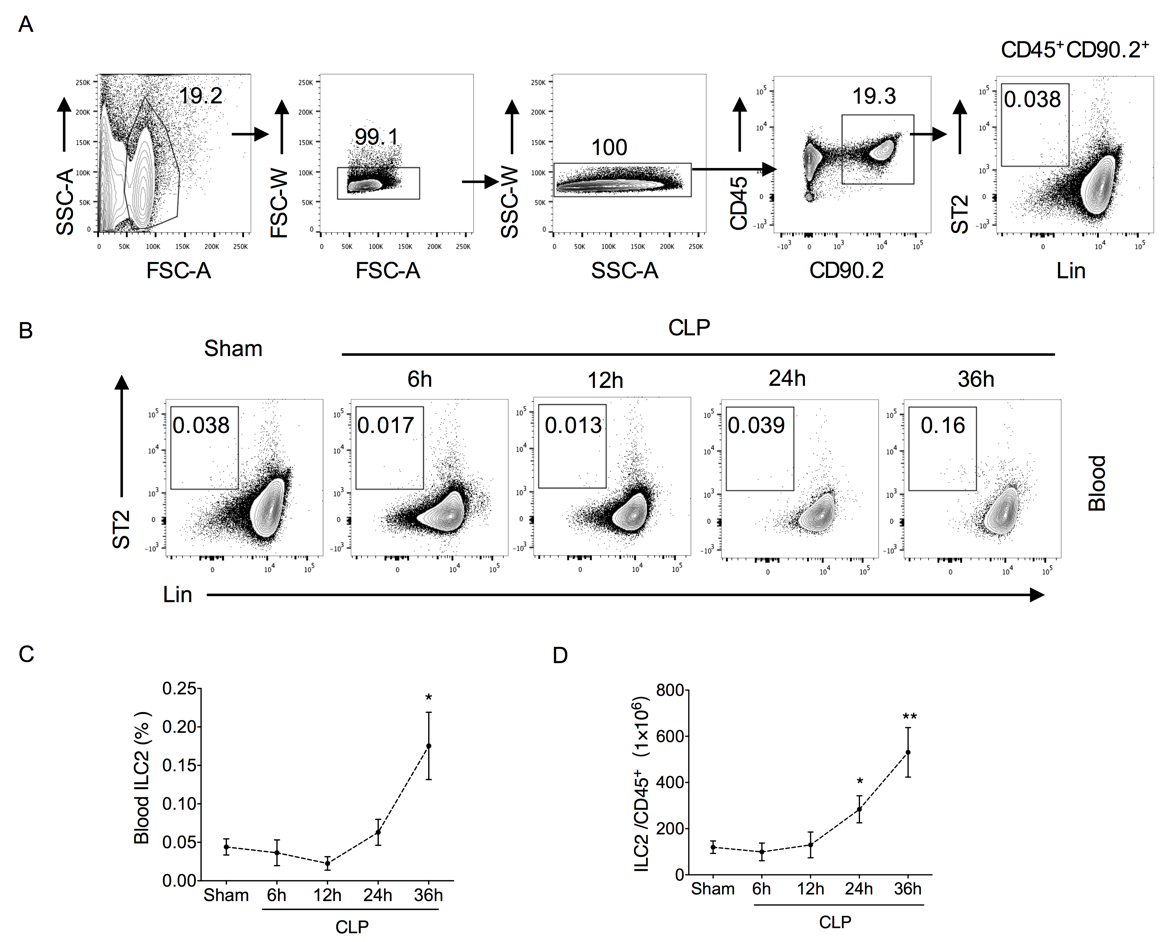


**Supplementary Figure E2. ILC2 in blood following sepsis of mice.**

(A) Representative flow cytometry plots and gating strategy of peripheral blood ILC2. ILC2 was defined as CD90.2+CD45+ Lin-ST2+ cells. A lymphocyte gate was drawn, and then CD90.2+CD45+ cells were gated into Lin-ST2+. (B) Representative flow cytometry plots showing percentages of ILC2 in peripheral blood of WT mice after sham surgery (36 h) and 6, 12, 24, and 36 h after CLP. (C) Line graph showing blood ILC2 frequency at time points up to 36 h after CLP. N>5 mice/group. (D) The absolute numbers of blood ILC2 at time points up to 36 h after CLP were calculated in every 1 × 106 CD45+ cells. Data shown as mean ± SEM. NS = not significant, *P<0.05, **P< 0.01.
